# Post-Infarction Left Ventricular Aneurysm Repair

**DOI:** 10.21470/1678-9741-2018-0188

**Published:** 2018

**Authors:** Paulo Roberto B. Evora

**Affiliations:** 1 Department of Surgery and Anatomy, Faculdade de Medicina de Ribeirão Preto da Universidade de São Paulo (FMRP-USP), Ribeirão Preto, SP, Brazil.

Dear Editor,

Kaya et al.^[1]^ presented a well conducted retrospective study including eighty-nine patients (74 males, 15 females; mean age 58±8.4 years; range: 41 to 80 years) underwent post-infarction left ventricular aneurysm repair and myocardial revascularization performed between 1996 and 2016. Ventricular reconstruction was performed using endoventricular circular patch plasty (Dor procedure) (n=48; group A) or linear repair technique (n=41; group B). In concordance with several published experiences, they concluded that the results of their study demonstrate that post-infarction left ventricular aneurysm repair can be performed with both techniques with acceptable surgical risk and with satisfactory hemodynamic improvement. I guess that this conclusion would cause a dangerous concept that both operative technique would be used independent of the aneurysm size.

**Fig. 1:**
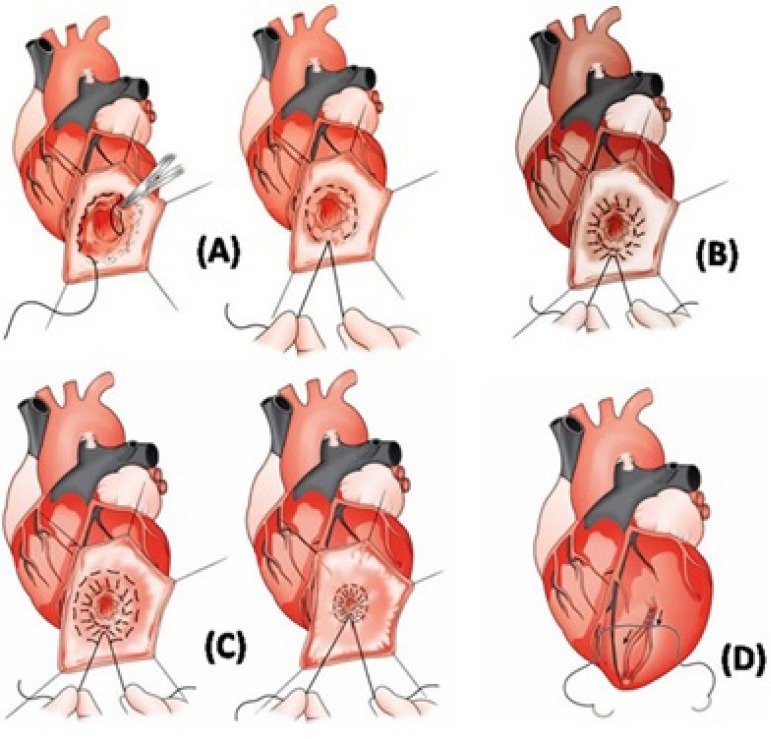
A) First endocardial encircling suture around the transitional zone between the scarred and normal tissue; B) Scar tissue plication using the same suture thread (this surgical maneuver keeps the aneurysm neck occluded, preserving the pyriform left ventricle shape); C) Second encircling suture is tightened, completing the aneurysm occlusion; D) The remaining scar tissue is oversewn with a running “out-out” suture, to ensure hemostasis^[3]^.

From this point of view, the linear repair technique can reduce the remained ventricular cavity. This situation inspired doctor Adib Jatene on his revolutionary concept of "geometrical reconstruction of the left ventricle" to treat aneurysms of this cardiac chamber, which is one of most important contribution of the Brazilian cardiac surgery^[2]^. Based on this concept we proposed a surgical variant technique to repair left ventricular aneurysms (Figure 1)^[3,4]^.

It is relevant to mention that there are, beside experiences around the world, convincing experiences for ventricular reconstruction: 1) Direct suture; 2) Modification of the Cooley technique with patch suture; 3) Dor patch plasty with septal exclusion^[5]^; 4) Jatene geometric reconstruction with semi-rigid bovine pericardial prosthesis, and 5) Attempts to compare different techniques without definitive proof of superiority among them. However, from safety and reduction of surgical time, the "no patch" surgical variants techniques would be useful for the decision whether to operate left ventricular aneurysm or akinesia^[3,5]^.

Doctor Kaya et al.^[1]^ pointed that "the decision on which technique to use in the repair was based on the size of the aneurysm during surgery and the extent of the scar tissue. In the case of smaller lesions without a marked aneurysmal sac, linear repair was preferred, whereas endoaneurysmorrhaphy was performed in case of larger lesions with a marked neck and fibrotic sac". This opportune observation *per se* is a clear introduction of considerable bias in comparative studies that need a great number of patients.
